# Tracking the effects of COVID-19 in rural China over time

**DOI:** 10.1186/s12939-020-01369-z

**Published:** 2021-01-14

**Authors:** Huan Wang, Markus Zhang, Robin Li, Oliver Zhong, Hannah Johnstone, Huan Zhou, Hao Xue, Sean Sylvia, Matthew Boswell, Prashant Loyalka, Scott Rozelle

**Affiliations:** 1 Freeman Spogli Institute for International Studies, Stanford University, 616 Jane Stanford Way, Stanford, California 94305 USA; 2 West China School of Public Health, Sichuan University, No. 17, Section 3 Ren Min South Road, Chengdu, Sichuan Province People’s Republic of China; 3 Gillings School of Global Public Health, University of North Carolina at Chapel Hill, 1101D McGavran-Greenberg Hall, CB#7411, Chapel Hill, NC 27599-7411 USA

**Keywords:** COVID-19, Rural community, China

## Abstract

**Background:**

China issued strict nationwide guidelines to combat the COVID-19 outbreak in January 2020 and gradually loosened the restrictions on movement in early March. Little is known about how these disease control measures affected the 600 million people who live in rural China. The goal of this paper is to document the quarantine measures implemented in rural China outside the epicenter of Hubei Province and to assess the socioeconomic effect of the measures on rural communities over time.

**Methods:**

We conducted three rounds of interviews with informants from 726 villages in seven provinces, accounting for over 25% of China’s overall rural population. The survey collected data on rural quarantine implementation; COVID-19 infections and deaths in the survey villages; and effects of the quarantine on employment, income, education, health care, and government policies to address any negative impacts. The empirical findings of the work established that strict quarantine measures were implemented in rural villages throughout China in February.

**Results:**

There was little spread of COVID-19 in rural communities: an infection rate of 0.001% and zero deaths reported in our sample. However, there were negative social and economic outcomes, including high rates of unemployment, falling household income, rising prices, and disrupted student learning. Health care was generally accessible, but many delayed their non-COVID-19 health care due to the quarantine measures. Only 20% of villagers received any form of local government aid, and only 11% of villages received financial subsidies. There were no reports of national government aid programs that targeted rural villagers in the sample areas.

**Conclusions:**

By examining the economic and social effects of the COVID-19 restrictions in rural communities, this study will help to guide other middle- and low-income countries in their containment and restorative processes. Without consideration for economically vulnerable populations, economic hardships and poverty will likely continue to have a negative impact on the most susceptible communities.

**Supplementary Information:**

The online version contains supplementary material available at 10.1186/s12939-020-01369-z.

## Background

China’s government issued nationwide guidelines to combat the COVID-19 outbreak in January 2020 and, after implementing strict lockdown measures for more than a month, gradually loosened the restrictions on movement in early March. These guidelines mandated social behaviors that were designed to prevent the spread of infection and were implemented by local health organizations [1]. Public service sectors, such as hospitals and schools, underwent major changes to accommodate these new guidelines [2]. In cities across the country, flights and train travel were limited, public events were cancelled or postponed, and schools were closed until further notice [3]. The rollout of these measures coincided with China’s Lunar New Year holiday—a time when a majority of the population returned to their homes to celebrate with family. In early March, as COVID-19 began to abate in all major city centers (with the exception of the Wuhan/Hubei epicenter), the restrictions on movement were gradually loosened. Controls on travel were lifted, and public transportation was resumed [4–6]. Schools in urban areas started to reopen in April [3].

Multiple studies were conducted on the effect that the COVID-19 restrictions had on employment in urban areas and found that most workers were still able to work from home during the quarantine. One study that used survey data from 64 cities shows that less than 2% of urban workers lost jobs [5]. Although approximately 25% of urban workers were not able to work as a result of the outbreak, measures were put in place by the local and national government to ensure that salaried workers in urban centers could not be laid off during this time [5]. China’s national government also implemented numerous incentives for large businesses in urban centers in an effort to achieve their goal of a quick economic recovery.

In addition to maintaining high employment rates, urban centers worked to mitigate the effects of these regulations on student learning by utilizing online learning and parental support. Research suggests that, although schools were uniformly closed in cities during the quarantine, urban children received online interactive instruction from teachers with support from parents [7]. Apart from livestreaming classes, the Ministry of Education worked with urban schools to develop an online learning platform to allow more than 50 million students and teachers to simultaneously connect and interact [8]. The supervision of urban parents also has been shown to play an important role in the success of in-home educational outcomes, as parents partnered with schools to support the autonomous learning process [9]. In urban schools, online teacher training also was made available (and often mandated); the training covered best practices for teaching online and how to navigate a variety of devices, and suggested the appointing of teaching technology consultants to minimize disruptions of technological errors [7]. Quality technology infrastructure and adequate network coverage, as well as familiarity with (and availability of) a variety of devices, made the shift from in-person to online learning relatively seamless in urban centers [7].

Unlike the straightforward transition to online learning seen in education, urban healthcare centers were faced with the challenge of accommodating a surge of residents who were seeking medical attention, all while sheltering in place. City hospitals faced an overflow of patients, particularly in the Wuhan epicenter, and had to turn away urban residents. To accommodate the overcrowding of medical centers, temporary hospitals, or *fangcang*, were erected in public spaces, such as community centers and public halls [10]. Research revealed that, for the majority of the urban populations, telemedicine and contact tracing were effective solutions to monitoring and slowing the spread of COVID-19 in major urban centers [11]. Using high-speed internet, the telemedicine system proved to be an effective tool in educating people about the virus as well as diagnosing, treating, and prescribing medicine to urban residents from their homes [12].

To help urban residents to cope with COVID-19, the government implemented various economic measures to keep businesses afloat and to mitigate income loss and unemployment. To minimize layoffs and offset income loss, financial institutions were encouraged to lend to small businesses through tax incentives [13]. In addition, relief measures were implemented in the form of tax extensions, which could be accessed via tax bureau websites and mobile applications. These extensions covered everything from real estate tax exemptions to social security payment deferrals [13]. To maintain employment, firms were not permitted to lay off salaried employees [14]. For those urban residents who were not covered by the employment guarantees, programs were implemented by the government to ensure that they were able to maintain a basic income. These programs not only simplified the unemployment application process but also extended the amount of time urban laid-off workers could receive unemployment benefits [15].

Although we have a body of literature that has allowed the world to study and follow the impacts on China’s residents in urban areas, there are still unanswered questions about how COVID-19 effected the 60% of the Chinese populace in rural communities [16]. What were the disease control measures, and how were they implemented in rural villages, especially those outside the epicenter of Hubei Province? What were the effects on employment, health care, and education? To what extent did the government provide aid to low-income families who, with no public safety net, were one of the most vulnerable subpopulations in China? Answers to these questions are important for not only understanding the welfare effects on rural communities, which make up more than half of the Chinese populace, but also the ability of rural residents to find employment, to continue learning, and to receive health care in the aftermath of the pandemic. These factors will play a key role in China’s overall economic recovery and future growth.

The goal of this paper is to document the quarantine measures implemented in rural China outside the epicenter in Hubei Province and to assess their socioeconomic effect on rural communities over time—both during the time that disease control measures were being strictly implemented and after their loosening. By examining the economic and social effects of the COVID-19 restrictions in rural communities, this study will help to predict China’s ability to recover economically as well as guide other middle- and low-income countries in their restorative processes.

## Methods

### Study design and participants

For this study, starting in late February 2020, we recruited village informants who took part in previous unrelated studies conducted by the research team. The previous studies covered 2069 villages in 540 townships across 60 counties in seven inland provinces: Ningxia, Shaanxi, Gansu, Jiangxi, Henan, Yunnan, and Sichuan. Together, the seven provinces account for over 25% of China’s overall rural population [17]. On average, the income per capita of rural residents in these provinces was 1683 USD in 2018 (ranging from 1127 USD per capita to 2185 USD per capita), slightly below the national rural average of 2209 USD per capita [18].

**Fig. 1:**
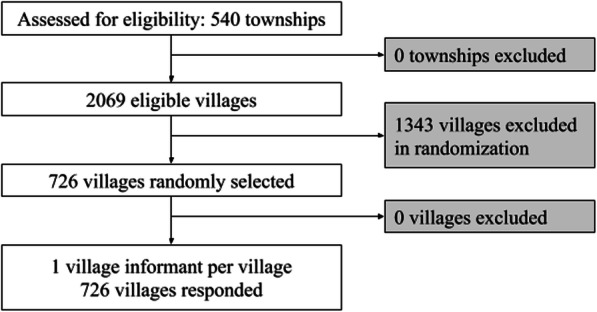
Flowchart of sample selection

To select a sample for this study, we first included all 540 townships. Within each sample township, we created a list of 10 randomly selected households and excluded individuals who lived in the local urban centers or county seats, leaving only those households in rural villages. We also excluded village officials, village doctors, and village teachers to ensure that our sample consisted of ordinary villagers. Finally, we made phone calls to the households on the list to complete the interview, with the goal to include 100 villages per province. In total, we randomly selected and interviewed 726 village informants, who resided in 726 randomly sampled villages. The sampling approach is summarized in a flow chart (Fig. 1). The number of counties, townships, and village informants for each province are listed in Table 1. We estimate that about 726,000 rural residents were represented in the sample villages.

**Table 1:** Sample distribution

Province	No. of sample counties	No. of sample townships	No. of sample villages	Avg. distance from village to county seat	Avg. no. of households in village	Han ethnicity (%)
Gansu	5	62	107	42.95	90	99
Ningxia	20	103	103	25.44	304	41
Shaanxi	11	99	105	39.70	168	100
Jiangxi	3	58	101	24.04	439	100
Sichuan	4	95	107	37.05	317	99
Henan	6	46	101	14.88	606	100
Yunnan	11	77	102	48.15	229	76
Total	60	540	726	33.17	308	88

The data presented in this paper were collected from three rounds of phone call surveys. The first round of the survey was conducted in late February 2020, when we interviewed all 726 village informants. In late March, prior to conducting second-round survey (approximately 1 month after the first round), we randomly selected 349 of the village informants who had completed the first round of survey. There were village informants from each county in the sample (on average, 6 village informants per county). The third round of survey took place in late April, and during this survey, we interviewed the remaining 371 of the village informants who had not been interviewed in late March. In total, 34 informants (out of 726 informants in total) had left their respective village for work by the time we conducted the second- and third-round surveys. We asked these 34 informants to refer us to a friend or family member who still lived in the village. Carried out in this way, there was little attrition in the second and third rounds of the survey.

### Data collection

During the three waves of the survey, we conducted three rounds of phone-call surveys to collect data from village informants during and after the quarantine. Each phone survey lasted approximately 1 h. During the survey, enumerators asked the village informants to characterize the nature of the quarantine and the consequences of the quarantine measures for the village as a whole. We informed village informants that their anonymity would be maintained and that they could answer the survey questions freely.

Each round of the survey questionnaire contained five sections. The first section collected information on the quarantine measures that were implemented, including transportation limitations, travel restrictions, and other rules, both within the village (that were implemented by local village leaders) and those taken by the government. The second section collected information about COVID-19 infections and deaths in the village and in the surrounding townships. The third section of the survey asked about the general effect of the quarantine on employment and income. The fourth section of the survey asked about the effect of the quarantine on the accessibility of education for rural students and about the nature of (non-COVID-19) health care in the village. The final section of the survey asked the village informant to describe how the national and local governments attempted to address negative consequences of the quarantine on rural villagers.

### Statistical analysis

Descriptive analysis was performed to compare the trends of control measures and effects reported by village informants. All analyses were done using Microsoft Excel and Stata 16 (Stata Corporation, College Station, TX).

## Results

### Quarantine measures

In February, China’s government implemented a number of strict quarantine measures across nearly all of the nation’s rural villages outside the epicenter (Table 2). Movement was restricted, with 87% of village informants who reported that they could not visit friends or family in other villages and 65% who reported that they could not leave their villages for shopping. Not only were villagers prevented from going on walks in 72% of the villages surveyed, but children were not permitted to play freely outside in 88% of the villages. Of the village informants, 96% reported that wearing a mask was mandatory to go outside, although only 16% of informants reported that surgical masks were readily available for purchase in their villages. Group activities were more strictly enforced than the movement of villagers: 99% of villages surveyed did not permit group entertainment activities, 98% did not permit weddings or funerals, and 97% did not permit villagers to visit other homes within the village.

**Table 2:** COVID-19 disease control measures reported in rural villages

Control measure	February *N* = 726	March *N* = 348	April *N* = 371
Visits not permitted from family or friends who live outside of the village	87%	17%	8%
Villagers not permitted to leave the village for shopping	65%	0.3%	0.3%
Villagers not permitted to go for walks	72%	14%	6%
Children not permitted to play freely outside	88%	21%	16%
Villagers required to wear masks to go outside	96%	89%	76%
Surgical masks available for purchase	16%	74%	97%
Group entertainment activities not permitted	99%	66%	50%
Weddings or funerals not permitted	98%	74%	55%
Villagers not permitted to visit other homes within the village	97%	53%	44%

Restrictions on movement were loosened in March and April (Table 2, Columns 2 and 3), but restrictions on mask wearing and group activities were maintained. In March, only 17% of village informants reported that their villages continued to ban visits to family or friends in other villages, with just 8% of informants who reported the same in April. During this period, only 0.3% of villages surveyed did not permit residents to leave the village for shopping. Similarly, restrictions on going for walks dropped to 14% of villages surveyed in March and then to 6% in April. By late April, only 16% of villages did not permit children to play freely outside. However, restrictions remained high on mask wearing, with 89% of village informants in March and 76% in April who reported that their village still required them to wear a mask to leave their homes. The availability of surgical masks, though, improved greatly over the availability in February, with masks available for purchase in 74% of villages in March and 97% of villages in April. Restrictions on group activities also were maintained. By the end of April, 50% of villages surveyed continued to prohibit group entertainment activities, 55% prohibited weddings and funerals, and 40% prohibited villagers from visiting and gathering neighbors.

### Infection rate and deaths

The three rounds of surveys revealed a low rural infection rate and zero deaths due to COVID-19 in sampled rural communities (Table 3). Only four of the 726 village informants reported COVID-19 infections in their villages in February. In total, there were only 10 individuals who were reported as being infected. One villager was reported as infected in two of the villages, two infections were reported in the third village, and six infections were reported in the fourth village. No further infections were reported in the March and April surveys. The sample of 726 villages represented about 726,000 rural residents, of whom 10 were infected. To the extent that our sample is representative of rural communities outside of the epicenter, this means that the implied infection rate for rural areas outside of Hubei Province was 0.001%. There were no COVID-19 related deaths reported in any of the villages for any of the three waves of the surveys.

**Table 3:** Effects of disease control measures on the spread of COVID-19

COVID-19 spread	February	March	April
Diagnosed patient in village	4/726 (0.55%)	0/348	0/371
Number of diagnosed patients per village^a^	2.5 (4.85–7.85)	0	0
Infection rate	10/726000 (0.001%)^b^	0	0

*Note.* Data are mean (95% CI) or *n*/*N* (%)

^a^Reported by four village informants

^b^Calculation based on 1000 villagers per village

To verify that the number of infection/death cases of COVID-19 that were reported by the village informants in the sample villages were valid, we crossed-checked information with official infection numbers released by national-, provincial-, and city−/county-level authorities. The cases reported by the four village informants in our sample were consistent with the official records.

### Employment and income

The initial February survey revealed widespread negative effects of the quarantine measures on employment in rural areas (Table 4, Column 1, Rows 1 to 5). Of the village informants, 74% reported that villagers had stopped working due to workplace closures. Travel to urban centers also was difficult, with 82% of village informants who reported that local public transportation had ceased operating and 64% who stated that villagers who owned vehicles were not permitted to drive themselves to cities. In addition to the issue of accessing transport to their urban destinations, 94% indicated that, within cities, rural individuals would not have been permitted to rent living quarters. Even if travel and accommodation were not issues, 67% of village informants stated that the fear of infection in their villages was so great that villagers did not want to leave the village to find employment.

**Table 4:** Reported effects of COVID-19 disease control measures on employment and income

Effect	February *N* = 726	March *N* = 348	April *N* = 371
Villagers unable to work because workplaces were closed	74%	40%	31%
Villagers unable to use public transportation to travel to city	82%	5%	2%
Villagers unable to drive or carpool to the city	64%	2%	0.1%
Villagers unable to rent a place to stay in the city	94%	10%	4%
Villagers decided not to leave the village to work due to fear of infection	67%	30%	22%
Villagers reported income decreased	92%	85%	91%
Prices of common goods were higher than last year	63%	66%	66%
Villages that received local government relief	N/A	17%	20%
Villagers received financial subsidies	N/A	9%	11%
Villagers received daily necessities	N/A	10%	11%

Although most of the quarantine measures that were keeping villagers from returning to work were lifted in early March, the March and April surveys revealed continued high levels of unemployment in rural areas (Table 4, Columns 2 and 3, Rows 1 to 5). By late March, only 5% of village informants reported an inability to use public transportation, and 2% reported restrictions on driving to cities. In addition, by late April, only 4% of informants reported restrictions that prevented rural workers from renting living quarters in cities. Despite the removal of these policy-erected barriers, village informants reported that 40% of villagers who worked last year were still not able to work in late March and that 31% of villagers were still not working by late April. Moreover, fear of infection continued to deter villagers from traveling to find work in 30% of villages in March and 22% in April.

**Fig. 2:**
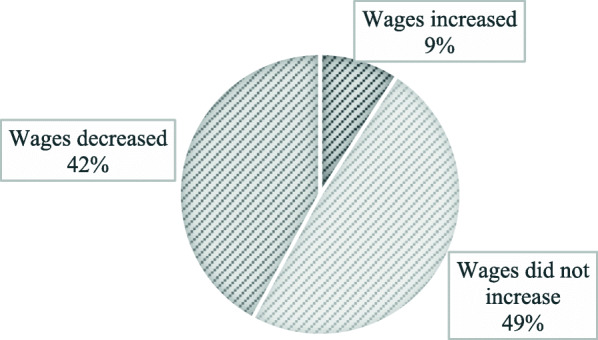
Percentage of village informants who reported an increase, no change or decrease in wages after work resumed

**Fig. 3:**
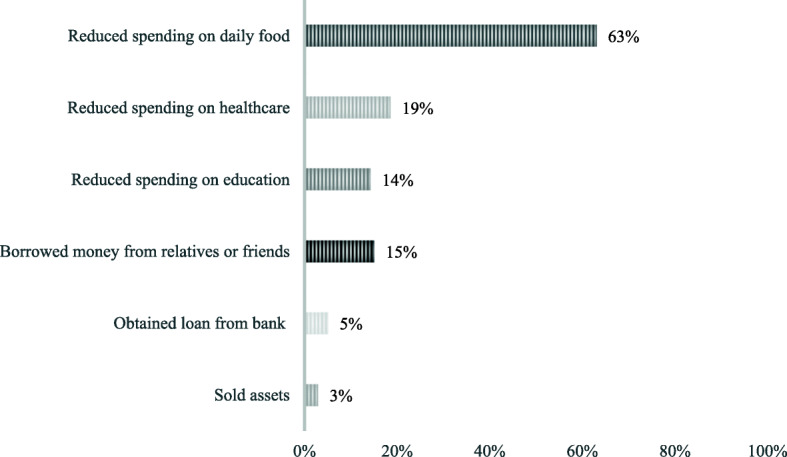
How villagers coped with decreases in income in April

High unemployment coincided with lower incomes and higher prices, with little aid from the government, leading rural villagers to cut daily expenditures (Table 4; Figs. 2 and 3). Village informants reported that income had decreased in 92% of villages in February, 82% in March, and 91% in April (Table 4, Row 6). In addition to lost income due to unemployment, 42% of village informants reported that rural workers who returned to work in April received decreased wages (Fig. 2). During this time period, prices rose drastically, with 63% of village informants who reported in February that the prices of common goods had increased from last year and 66% who reported the same in March and April (Table 4, Row 7). Moreover, most villagers did not benefit from government relief, with only 20% of village informants who reported the existence of relief programs in their villages by late April (Rows 8 to 10). Of those that who receive government relief, 11% of villages reported that few villagers received small financial subsidies (500 RMB, equivalent of 127 USD); 11% of village informants said that there were families in their communities who received daily necessities, such as rice, oil, and seeds. As a result, villagers coped by cutting their expenditures on daily necessities (Fig. 3). In April, 63% of village informants reported that residents of their villages spent less on food, 19% reported less spending on health care, and 14% reported less spending on education.

### Education

Village informants reported pervasive disruptions to regular schooling as well as various efforts over time by teachers and policymakers to mitigate these disruptions (Table 5, Rows 1 to 5). In the villages surveyed, no schools had been reopened in February or March, and only a quarter had reopened by late April. During the time that schools were closed, however, the proportion of local teachers who assigned daily homework to students increased from 69% in February to 91% in April. Among these teachers, the proportion who regularly graded homework and provided feedback rose from 83% in February to 96% in April. In addition, the ratio of schools that organized online learning climbed from 71% in February to 89% in April.

**Table 5:** Reported effect of COVID-19 disease control measures on education

Effect	February	March	April
School in session	0/726 (0%)	0/348 (0%)	91/371 (25%)
Mitigating efforts by school districts			
Local teachers assigned homework for students daily	500/726 (69%)	302/348 (87%)	337/371 (91%)
Local teachers corrected homework for students daily	415/500 (83%)	284/302 (94%)	325/337 (96%)
Schools organized online courses	513/726 (71%)	304/348 (87%)	329/371 (89%)
Challenges for rural students learning online			
Online courses were not taught by local teachers	307/513 (60%)	117/320 (37%)	91/329 (28%)
Teacher could not see video of students during online courses	413/513 (81%)	240/304 (79%)	210/329 (67%)
Devices students used to get online			
Phones	437/513 (85%)	280/304 (92%)	318/329 (97%)
Computers	81/513 (16%)	58/304 (19%)	103/329 (31%)
Televisions	212/513 (41%)	131/304 (43%)	130/329 (40%)
Internet connection were often not stable	350/513 (68%)	232/304 (76%)	256/329 (78%)
Students needed to go outdoors to maintain connection	N/A	92/304 (30%)	52/325 (16%)
Reported negative effect on education	570/726 (79%)	293/348 (84%)	337/371 (91%)

**Fig. 4:**
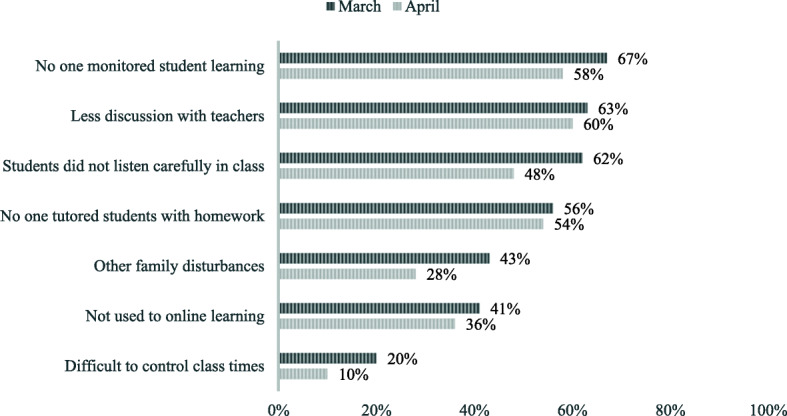
Problems that disrupted the quality of online learning, as reported by village informants

Despite the efforts made to mitigate learning disruptions, our data also show that there were factors that almost certainly limited the quality of online learning in the rural communities. For example, numerous online classes were not taught by local teachers, meaning that online instructors were teachers with whom students were unfamiliar. These teachers also may have taught the curriculum at a pace which was faster than rural schools could keep up with. Non-local teaching was 60% in February and changed to 37% in March and 28% in April (Table 5, Row 5). During the online classes, although 75 and 95% of students in February and April were able to see their teachers in online classes, only 19% of teachers in February and 33% in April could see their students (Row 6). Around 90% of students used phones to connect to online classes, whereas less than a fifth used computers (Rows 7 and 8). The proportion of those who used television to tune into educational broadcasts was consistent, at around 41% (Row 9). The percentage of village informants who reported that students in their communities suffered from internet connectivity issues rose from 68% in February to 78% in April, to the extent that roughly a third of students had to stay outdoors to maintain reception (Row 10). Moreover, village informants reported numerous problems that they believed disrupted the quality of online learning (Fig. 4). In April, 60% of village informants mentioned that there was less discussion between students and teachers, 58% noted that no one monitored student learning, 48% indicated that students did not listen carefully in class, 54% stated that nobody guided students with homework, and 28% noted that there were other family disturbances. These factors contributed to the increasingly negative feelings toward the impact of control measures on the education of local children, from 79% in February to 84% in March and 91% in April (Table 5, Row 12).

### Health care

Most villages reported that health care remained accessible during and after quarantine (Table 6, Rows 1 to 3). In February, 71% of the village clinics were open daily. After the quarantine, the share of village clinics that reported being open daily increased to 91% in March and then 98% in April. Almost all of the village informants reported that they were able to leave the village to seek health care in February during the strictest period of the quarantine (95%) and after the easing in March (84%) and April (96%). Medicines were reported to have been generally available in most of the sample villages during the quarantine in February (89%) and after the quarantine in March (90%) and in April (98%).

**Table 6:** Reported effect of COVID-19 disease control measures on health care

Effect	February	March	April
Villagers were able to see a doctor outside of village	687/726 (95%)	291/348 (84%)	355/371 (96%)
Village clinic was currently open	516/726 (71%)	315/348 (91%)	362/371 (98%)
Villagers were able to buy medicine	645/726 (89%)	313/348 (90%)	362/371 (98%)
Villagers chose to delay health care due to COVID-19	N/A	68/348 (20%)	39/371 (11%)
Villagers knew how to use online doctors/telemedicine	N/A	14/348 (4%)	12/371 (3%)
Negative impact on health care	62%	45%	44%

Although health care was generally accessible, about 20% of the village informants reported that people had delayed seeking routine health care due to COVID-19 in March (Table 6, Rows 4 to 6). Later in April, the number of villagers who delayed their health care due to COVID-19 decreased to around 11%. Few villagers reported being aware of telemedicine services in March (4%) and April (3%). Overall, 62% of the village informants reported a negative impact of COVID-19 on health care in February, 45% in March, and 44% in April.

## Discussion

### Quarantine measures

The findings of our surveys suggest widespread enforcement and compliance with quarantine measures across rural China during and after the quarantine period. In February, virtually no village informants reported being able to move freely or gather in groups. Even with the lifting of restrictions in March, village authorities remained cautious and kept in place certain measures throughout April. For instance, approximately half of the rural villages surveyed in April still did not permit group activities, with 44% of villages that banned visiting neighbors from playing cards. Children also were not allowed to play freely outside in about a sixth of villages surveyed in April. These restrictions were kept despite virtually no increase in China’s COVID-19 case count throughout April [19].

The high level of compliance with quarantine measures in rural areas reflects the capacity of China’s government to impose and enforce community restrictions. In this way, the efforts in rural China were much like those in urban China, where local health administrative departments were in charge of implementing strict quarantine measures [1]. In the case of rural areas, village informants explained that village party committee members, doctors, and village volunteers worked together as “epidemic prevention and control teams” to enforce quarantine measures, such as mask wearing [20]. Everyone who went in and out of the village had to register at a station where body temperature was checked. As is evident from our survey data, the widespread compliance of rural communities is likely due to the presence of strong local government organizations and communication. The enforcement of similar quarantine measures is likely not possible in most other middle- and low-income countries, where strong and widespread local governments are lacking.

### Infection rate and deaths

Our surveys demonstrate that strict quarantine measures throughout rural China coincided with low infection rates and indicate that likely no widespread cover-up of COVID-19 cases occurred. Only 10 cases were reported in total in our February survey, and were all isolated and treated in designated hospitals. No village informant reported any deaths from the virus. The implied infection rate in our sample area was about 14 infections for every 1 million people. This infection rate aligns with the official rate of 11 infections per million people reported for all areas of China (except for Hubei Province).

One key question, of course, is whether the information from the village informants was accurate and truthful. We believe so for three reasons [21]. First, information generally spreads quickly in villages in China [22]. Despite restrictions on physical movement in villages, nearly ubiquitous mobile phone and social media use would have allowed news of infection cases to spread quickly [23]. If an infection were present in a village, relatives and neighbors in the village would certainly have known. Second, to our knowledge, there was no attempt to keep infections a secret from residents. Rather, informants described how village authorities made efforts to ensure that all village residents were aware of infections through methods such as loudspeaker announcements and large banners. Finally, during the interviews, the research team’s enumerators unilaterally stated that almost all village informants were willing to openly share with us their knowledge of infections in their villages.

Were the strict quarantine measures responsible for the low levels of infection in rural communities outside the epicenter? In the absence of counterfactual information, it is not possible to know for certain whether the disease control measures described in the previous section are directly responsible for the low rural infection rates in our sample. Although we do not have causal data between strict control measures and the limited spread of COVID-19 in rural areas, our survey demonstrates that the two events coincided with one another. When considering this in conjunction with the experience of nations such as the United States, Brazil, and India, where fewer control measures were accompanied by greater virus spread [24], it is highly likely that the restrictive and widespread control measures indeed resulted in the reported low infection rates.

### Employment and income

The implementation of quarantine measures resulted in a radical increase in unemployment in China’s rural areas that persisted even 2 months after the measures were relaxed. Nearly three-quarters (74%) of rural workers outside of Hubei Province who were employed a year ago were unemployed in February as a result of workplace closures and layoffs related to COVID-19. Our surveys show almost one-third (31%) of all rural workers were still unemployed in late April.

Such high levels of unemployment stand in stark contrast to the unemployment rates announced in China. As a result of the pandemic and subsequent control measures, China’s official unemployment rose by less than 1%, from 5.3% in January to a peak of 6.2% in February [25]. Even though the rate of 5.9% (as of May 2020) was higher than pre-pandemic levels, the slight overall change implies that the effect of the quarantine measures on China’s economy was insignificant, and it would seem to be likely to completely recover. It is important to note, however, that China’s reported unemployment rates applies only to urban areas and has always excluded rural residents and seasonal migrant workers [26], the same groups who did not receive protection from layoffs during the pandemic. As a result of this omission, rural unemployment is largely overlooked. Thus, our surveyed unemployment rate differs greatly from the official rate and shows that nearly one out of three (31%) rural workers who had jobs in 2019 were still not working as of late April, suggesting that quarantine measures were having a larger, long-term negative effect on China’s economy than what official figures reveal.

What are the factors that influence the high rates of unemployment? Apart from workplace closures and layoffs related to COVID-19, rural workers also could have ceased working due to transportation difficulties. Fear of infection (which was still a deterrent in a fifth of the villages in April) played a significant role in villagers’ decision-making processes (see Additional file 1), resulting in an unemployment rate even higher than our estimates. Another likely factor that contributed to the high rural unemployment rate was weak domestic and international demand as China’s consumers coped with lost wages and other countries coped with the global recession [27, 28], leading the nation’s factories to continue operating at lower capacity and hiring fewer workers (at lower wage rates). Rural unemployment/income is, therefore, likely to remain high as demand stays low and workers struggle to find jobs, resulting in unused human capital and hindering China’s economic recovery.

Disaggregated data on the rate of economic recovery that has been published in the literature support the findings in this study of the slow recovery that is being borne in no small part by rural workers. A March survey by Peking University, with data from over 1 million enterprises, showed that job listings for lower-salaried workers (below 4000 RMB per month) dropped by 44% compared to the same time last year [29]. In contrast, the drop in postings for jobs that were in the higher salary range (> 15,000 RMB) was only 12%. This finding validates the results of our survey and suggests that the recovery has been slower for low-wage workers, the majority of whom are rural and did not enjoy protection from layoffs.

The high economic toll of the high rural unemployment rate due to COVID-19 is evident when considering the amount of lost wages of rural workers during and after the quarantine period. In normal times, China has 288 million migrant workers who leave their counties for extended periods to work in distant cities and an additional 93 million workers who live in their villages but work within their own counties [17]. Together, these two populations of rural workers amount to 381 million people. According to our data on unemployment due to workplace closures, it is assumed that 74% of these workers were unemployed in February, 40% in March, and 31% in April. Rural migrant workers make an average of 560 USD per month [30], but they are paid only if they work. Altogether, this means that the lost wages of rural workers over the three-month period amount to at least 309 billion USD. This value for lost wages does not even account for the lower wages received by 42% of rural workers who had returned to work by April. In comparison, the highest estimate for the global economic cost of the SARS outbreak in 2003 was only 100 billion USD [31].

Although rural Chinese residents likely bore the brunt of the negative economic effects of the quarantine, they have received little assistance from the government. According to the literature and official reports, there have been a number of government relief measures that have benefited urban areas, including subsidies for key enterprises, extensions on tax payment deadlines, and emergency loans to qualifying firms [13]. This is despite the fact that government incentives for companies allowed urban salary-earning workers to continue to be paid, protected from layoffs, and able to rely on strong social support programs if needed [32]. In contrast to the widespread government aid that urban areas received, by April, only 20% of village informants reported that their villages had received any government relief. A mere 11% of the villages surveyed received any financial subsidies, which averaged only about 127 USD—not even a quarter of a migrant worker’s average monthly salary [30]. Another 11% of government relief measures distributed necessities, such as rice, oil, disinfectant, and seeds, but it likely had little long-term impact on the financial situation of village families.

Without supplemental income from government relief measures, the multiple months of lost wages will likely have long-lasting effects on rural households. In rural China, the average monthly income of a migrant worker is 560 USD, while the per capita monthly disposable income of a family that relies fully on farming is only 190 USD [30, 33]. Thus, with prolonged high unemployment and decreased income for rural migrant workers, it is expected that rural households have lost (and will continue to lose) a large portion of their annual income. Coupled with the increased price of common goods, households have been forced to cope by cutting back spending on daily necessities, such as food and health care. The decrease in food expenditures in 63% of surveyed villages is particularly concerning because it is likely that rural families replaced relatively expensive meats and fruits in their diets with grains and starch, resulting in less dietary diversity and possible micronutrient deficiencies. Rural children already face high levels of anemia from poor nutrition; thus, these additional nutritional deficiencies could worsen this issue and negatively affect children’s long-term educational outcomes [34]. It is apparent that quarantine measures could have long-lasting negative effects on rural families.

### Education

Our results show that, despite nationwide efforts to mitigate learning disruptions, the quarantine may have had a negative impact on the educational outcomes of rural children. China is one of the few countries where the existence of internet connectivity in remote rural areas made long-distance learning a practical possibility during the quarantine [35, 36]. Nevertheless, internet connectivity and infrastructure issues were a major barrier for many. Many households also did not have appropriate electronic devices for online learning. Instead, a large share of students had to use phones for online learning, and few used computers. Finally, parental assistance in learning often was not seen in rural communities. More than half of the village informants reported that no one monitored rural students when they learned or tutored them regarding homework, and they found that these problems disrupted the quality of online learning. Further, unlike our employment results, where efforts to mitigate unemployment were ongoing throughout the quarantine, we found little improvement in our results on education over time. Because roughly 423 million students across urban and rural China learned online for at least 2 months during school closures, it is likely that access to quality online learning will continue to play a major role in determining educational outcomes [37].

Our study also demonstrates that the negative impact of the quarantine on education had a number of barriers to learning. For example, under quarantine measures, there appears to be a severe lack of teacher-student interaction. Teacher-student interactions have been shown to be essential for student learning [38, 39]. Online classes are sometimes led by teachers who have never met their students previously, so those teachers are unable to accurately determine the learning nuances of each student, and, therefore, the effectiveness of their interactions would likely decrease. When local teachers were teaching, they could not even see students in their online classes, which impedes teacher-student interactions through video calls. As a result, about a half of the students in our survey turned to asynchronous learning by watching educational television broadcasts, for which students were told to tune into a specific channel to watch pre-recorded lecture videos. Research has shown, however, that pre-recorded online instruction has zero or even negative effects on learning, which poses an additional challenge to educational growth [38, 40].

Although the low levels of teacher-student interactions are naturally associated with online, in-home learning, our data show that there were other issues, such as those related to family, that put rural students at a greater disadvantage. Family disturbances might even have actively harmed the children’s education. Most rural grandparents, and even some rural parents, have limited educational attainment and are not sufficiently familiar with online learning technologies. This greatly limits their ability to help their children with online schoolwork [41, 42].

When comparing the experience of rural students to students from urban areas in the provision of online, in-home learning, it is almost certain that the COVID-19 pandemic has further widened the rural-urban education gap. As discussed in the first part of this paper, urban students likely maintained quality in-home learning that resulted from greater access to suitable learning devices and parental supervision [9]. To the extent that the smoother and more supported efforts in urban schools compared to the experience of rural students (as shown in this paper), there is no reason to think that the inequalities in rural-urban schooling have diminished during the winter and spring of 2020.

### Health care

The good news is that most villagers in rural China had access to health care during and after quarantine. Throughout the 3 months in our survey, almost all villagers were able to leave the village to seek health care. Moreover, even during the quarantine period, most village clinics were open. By the end of the quarantine period, almost all clinics were back in operation. During and after the quarantine, medicine remained accessible for purchase.

Despite the accessibility of rural health care, recent research in China that focuses on the quality of rural health care suggests that the benefits of the severe quarantine measures and the associated low rates of infection cannot be overestimated. Village and township doctors in rural areas have been shown to misdiagnose and mistreat patients at startlingly high rates [43]. Although diagnosis and treatment of COVID-19 would likely have been better given higher levels of awareness, a healthcare system staffed by poorly trained clinicians suggests that the rural healthcare system would have been ill-prepared to handle a large-scale outbreak. As such, it is fortunate that the infection rates were low in rural communities.

Although the rural healthcare system did not break down during or after the quarantine, the health of rural residents was still negatively affected due to villagers’ choosing to delay their health care. Even after quarantine measures were lifted, 11% of villagers still chose to delay their health care. If we assume a similar rate across all rural populations in China, that means that, even 2 months after the quarantine was lifted, there are still 61 million people who delayed their health care. This could lead to many serious health complications other than COVID-19 [44]. The delay of health care may have been due to COVID-19 measures, which include restrictions on travel and fear of infection. Indeed, over time, when the pandemic became less severe, more people decided to seek medical care. Research has documented the same pattern of delayed health care in urban China [45, 46].

## Conclusion

According to our survey of 726 randomly selected rural villages during and after the strict COVID-19 quarantine measures were enforced, these measures likely aided the containment of infection in rural China, resulting in a low infection rate. Nevertheless, there were very high costs for rural areas under quarantine. A sharp increase in rural unemployment persisted for nearly 2 months after the measures were relaxed, suggesting that the rate of economic recovery was not as clear and rapid as China’s government indicated. Although transportation and rental restrictions were largely lifted by March, there were fewer employment opportunities due to lower demand inside and outside of China. In addition, rural workers in some villages were reluctant to leave their village and return to work due to fear of infection. Few state government relief measures have been directed at the rural populace, and with the reportedly rising prices of common goods and nearly 2 months of lost income, rural households had to cut down on food and health expenditures.

Beyond the effects on employment, the virus affected villagers in other ways. Although local governments and school systems have made great efforts to minimize disruptions to learning by implementing online classes, policymakers have largely neglected the inaccessibility of the internet infrastructure in rural villages, household electronic devices, and educational software in rural regions. There were also many families who reduced their expenditures on health care, an act that has had a cost that, to date, has not been measured.

The pandemic should induce China to put more effort into the healthcare system in rural communities. The poor quality of rural clinicians could have left rural areas ravaged if the infections had spread to rural communities. Indeed, the strict quarantine measures in rural China prevented an epidemic in rural areas where primary care would be largely ineffective against surges in case numbers. In preparation for the next outbreak, efforts to improve China’s rural healthcare system, particularly in primary care, could be critical to thwarting catastrophic effects on rural populations in the future.

As COVID-19 continues to spread throughout the globe, our findings are increasingly relevant for other low- and middle-income countries. Workers around the world face huge losses of income in the coming weeks and months. As governments implement quarantine measures, they must also consider the needs of economically and socially vulnerable communities or face dramatic increases in economic hardship and poverty among the hardest hit.

## Supplementary Information

**Additional file 1.**

## Data Availability

The dataset used and/or analyzed during the current study are available from the corresponding author on reasonable request.
