# Supplementary material for: Tracking the effects of COVID-19 in rural China over time
Source: Int J Equity Health. 2021 Jan 14;20:35. doi: 10.1186/s12939-020-01369-z (PMC7807215; doi:10.1186/s12939-020-01369-z)

**Supplemtary Information**

***Additional File 1***

To further compare the relative magnitude of the relationship between various COVID-19 control measures and the reported unemployment rate in the village, we conducted a multivariate ordinary least squares (OLS) regression model. The basic specification of the model is:

$Y_{i}=\alpha C_{i}+\beta_{1}F_{i}+\varepsilon_{i}$ (1)

where Y*i* represents the dependent variable, which is the unemployment rate reported in village *i*. C*_i_* is a vector of COVID-19 control measures in village *i*. These control measures include highway blockages (equals 1 if villagers were unable to use the highway to travel to the city), public transportation restrictions (equals 1 if villagers were unable to use public transportation to travel to the city), interpersonal interaction limits (equals 1 if visits were not permitted from family or friends who live outside of the village), group activities control (equals 1 if group activities were not permitted). F*_i_* is a vector of village factors in village *i*, including the percentage of villagers who decided not to leave the village to work due to fear of infection, diagnosed COVID-19 patient in the village (equals 1 if yes), and village clinic closure (equals 1 if yes). $\varepsilon$*_i_* is a random error term.

The estimated results from the OLS regression model (see Appendix Table 1) show that the reported unemployment rate in the village was significantly associated with the decision of villagers not to leave the village due to fear of infection. The results also demonstrate that the unemployment rate was not significantly associated with other COVID-19 control measures (such as highway blockages, public transportation restrictions, or interpersonal interaction limits). The correlation coefficient between reported unemployment and fear of infection is 0.55, meaning that a one-percentage-point increase of villagers who decided not to leave the village to work due to fear of infection correlates with about a half-percentage-point increase in unemployment even after controlling for other measurable factors in the village. Although there is a positive correlation between reported unemployment rate and COVID-19 control measures, such as highway blockage, public transportation restrictions, group activity control, had a diagnosed patient in the village, and village clinic closure, none of these correlations is statistically significant (at *p*-value < 0.01).

Although our empirical findings cannot reveal precisely why, there are several reasons that rural villagers decided not to leave their villages for work due to a fear of infection. First, rural households in China have a weaker social safety net compare to their urban peers, with limited access to catastrophic medical insurance [1]. In addition, rural residents who had already moved more permanently to urban areas to live and work have a higher probability of being covered by China’s medical insurance system [2]. Further, because overall income (and wealth) of rural residents is lower than that of urban residents, the share of healthcare expenditures in their per capita disposable income is nearly twice as high [2]. Finally, the health care provided by the village clinics in rural areas has been shown to be of poor quality [3,4]. Hence, rural residents may be more afraid of being infected by the virus and having to rely on poor-quality care. All of these factors combined may make the threat of COVID-19 infection relatively higher to the share of the population in rural China who decided not to migrate to the urban areas for work.

As with the descriptive statistics, caution should be taken when interpreting the estimated findings from the OLS regressions, especially because COVID-19 affected almost all villages in rural China, and thus there was not a lot of natural variation to help identify causal effects. In addition to the village factors we included in our model, rural employment might be largely affected by the overall economy. Other factors, such as weak domestic and international demand as China’s consumers were trying to cope with lost wages and the overall recession [5,6], are likely to also contribute to the high rural unemployment rate. Unfortunately, in this study, our village-level data could not capture these external factors.


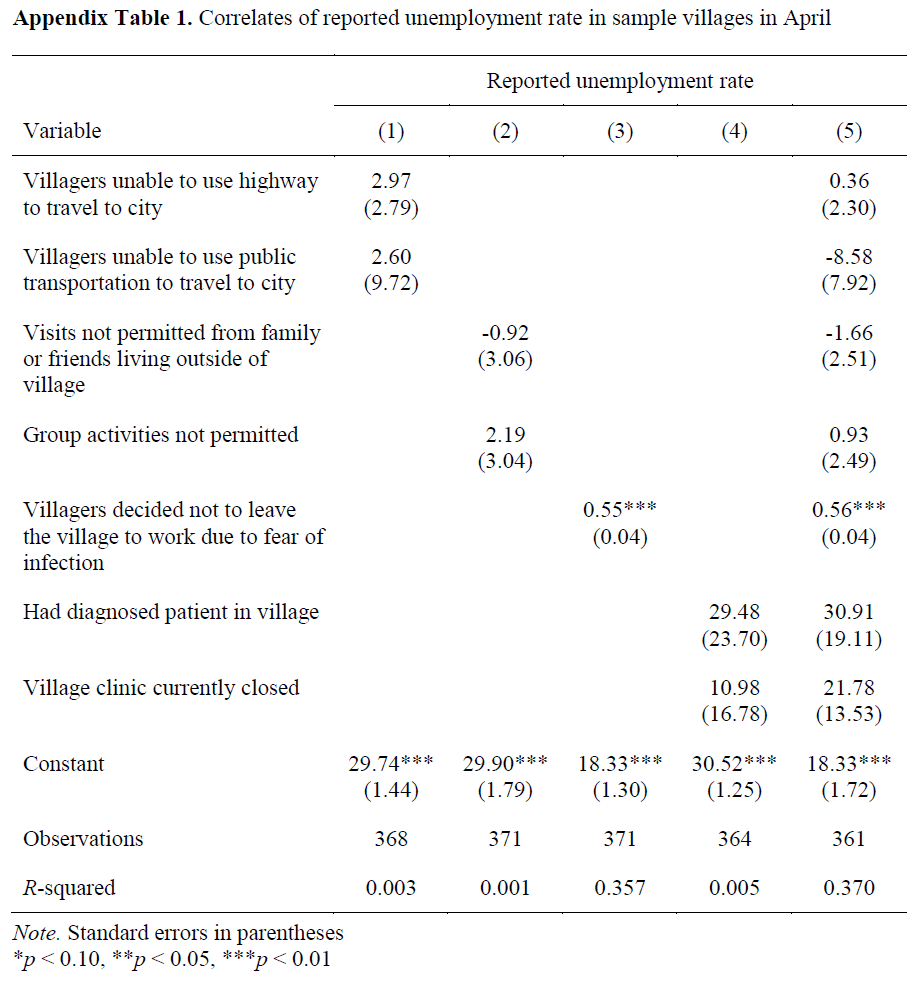

Supplement: Supplementary file 1 — Additional file 1. [file 12939_2020_1369_MOESM1_ESM.docx]
